# Effect of circumferential resection margin status on survival and recurrence in esophageal squamous cell carcinoma with neoadjuvant chemoradiotherapy

**DOI:** 10.3389/fonc.2022.965255

**Published:** 2022-09-02

**Authors:** Yi-Min Gu, Yu-Shang Yang, Wei-Li Kong, Qi-Xin Shang, Han-Lu Zhang, Wen-Ping Wang, Yong Yuan, Guo-Wei Che, Long-Qi Chen

**Affiliations:** ^1^ Department of Thoracic Surgery, West China Hospital of Sichuan University, Chengdu, China; ^2^ Department of Otolaryngology, Head and Neck Surgery, West China Hospital of Sichuan University, Chengdu, China

**Keywords:** neoadjuvant chemoradiotherapy, circumferential resection margin, prognosis, recurrence, esophageal squamous cell carcinoma,

## Abstract

**Background:**

The aim of this study was to investigate whether circumferential resection margin (CRM) status has an impact on survival and recurrence in esophageal squamous cell carcinoma after neoadjuvant chemoradiotherapy.

**Methods:**

We screened patients with esophageal squamous cell carcinoma who underwent esophagectomy from January 2017 to December 2019. The CRM was reassessed. Patients were grouped into a CRM of 1 mm or less (0 < CRM ≤ 1 mm) and a CRM greater than 1 mm (CRM>1 mm). The impact of CRM on survival was investigated using Kaplan–Meier analysis and Cox regression modeling. The optimal CRM cut point was evaluated using restricted cubic spline curve.

**Results:**

A total of 89 patients were enrolled in this study. The CRM status was an independent risk factor for the prognosis (HR: 0.35, 95% CI: 0.16-0.73). Compared with a CRM of 1 mm or less, a CRM greater than 1 mm had better overall survival (HR: 0.35, 95% CI: 0.16-0.73, log-rank *P* = 0.011), longer disease-free survival (HR: 0.51, 95% CI: 0.27-0.95, log-rank *P* = 0.040), and less recurrence (HR: 0.44, 95% CI: 0.23-0.85, log–rank *P* = 0.015). We visualized the association between CRM and the hazard ratio of survival and identified the optimal cut point at 1 mm.

**Conclusions:**

A CRM greater than 1 mm had better survival and less recurrence compared to a CRM of 1 mm or less. A more radical resection with adequate CRM could benefit survival in patients with esophageal squamous cell carcinoma after neoadjuvant therapy.

## Introduction

Esophageal cancer is the seventh most common malignant tumor and the sixth leading cause of cancer-related deaths worldwide (1, 2). To date, neoadjuvant chemoradiotherapy plus surgery has formed the standard treatment for local advanced esophageal cancer based on the CROSS study and the NEOCRTEC5010 study (3, 4). However, the recently published 10-year outcomes of the CROSS study have shown that 49% of patients had overall disease progression in the neoadjuvant chemoradiotherapy plus surgery group (5). The prognosis for patients with esophageal cancer remains unsatisfactory.

The prognostic significance of involved circumferential resection margin (CRM) in esophageal cancer has long been recognized (6, 7). However, the optimal CRM criteria in esophageal cancer remains controversial (8). Brac et al. found that tumor circumferential resection margins > 0 mm (standard from the College of American Pathologists) were considered adequate (9). On the contrary, Rao et al. found that a circumferential resection margin greater than 1 mm (standard from the Royal College of Pathologists) was more accurate for predicting prognosis (10). In addition, although neoadjuvant therapy improves the probability of R0 resection, induced fibrosis may contain a small amount of minimal residual disease (11). Neoadjuvant therapy has been reported to affect the CRM cut point (12). Therefore, the optimal CRM definition after neoadjuvant therapy warrants more attention.

The aim of this study was to investigate whether a CRM greater than 1 mm (CRM>1 mm) has an impact on survival and recurrence compared with a CRM of 1 mm or less (0 < CRM ≤ 1 mm) and to attempt to determine the optimal CRM in esophageal squamous cell carcinoma with neoadjuvant chemoradiotherapy.

## Method

### Study population

We reviewed our prospectively collected database for consecutive patients who underwent curative esophagectomy following neoadjuvant therapy at West China Hospital of Sichuan University from August 2017 to April 2019. The information collected included patient demographics, neoadjuvant chemoradiation therapy, perioperative outcomes, pathology, and follow-up data. Inclusion criteria included (1) locally advanced esophageal squamous cell carcinoma; (2) receiving esophagectomy following neoadjuvant chemoradiotherapy; (3) ypT3; and (4) at least 15 lymph nodes should be removed and assessed to achieve adequate nodal staging. Exclusion criteria included (1) CRM = 0; (2) adenocarcinoma; (3) coexistence of other malignancies; (4) positive proximal or distal margin; and (5) salvage surgery. Ethics approval for this study was granted by the Ethics Committee of West China Hospital, Sichuan University (No. 2022269), and informed consent was waived.

### Neoadjuvant chemoradiotherapy

Perioperative tumor staging was based on endoscopy, percutaneous neck ultrasound, endoscopic ultrasound, bone scan, and magnetic resonance imaging of the brain. All patients had either contrast-enhanced computed tomography or positron emission tomography-computed tomography. Neoadjuvant chemoradiotherapy was administered as a standard treatment for locally advanced ESCC. The recommended neoadjuvant chemotherapy regimens included paclitaxel plus cisplatin or 5-fluorouracil plus cisplatin in combination with 45 Gy of concomitant radiotherapy.

### Surgery

After 2–4 cycles of neoadjuvant regimens, ESCC patients were recommended to undergo a preoperative assessment to determine the feasibility of the operation. Minimally invasive McKeown esophagectomy was performed in all patients. En-bloc esophagectomy and complete two-field lymph node dissection was standard. Three-field lymph node dissection was only performed in patients with highly suspicious cervical nodal disease. Cervical esophagogastrostomy was performed using hand-sewn double layer sutures. The same team of surgeons performed all operations, using similar surgical techniques and following uniform postoperative care.

### Circumferential resection margin assessment

The specimen was painted with Indian ink on the outside, fixed, and then sectioned transversely to examine the distance between the tumor and margin (7). The circumferential resection margin was defined as the distance between the surgical margin and the nearest tumor edge. The features of the tumor, lymph node involvement, lymphovascular invasion, and perineural invasion were reexamined by two independent pathologists. Tumor regression was graded using the modified Ryan scoring system (13). The 8th edition of the American Joint Committee on Cancer TNM staging system was used to determine the pathologic stages.

### Follow-up

Data on locoregional recurrence, distant recurrence, and survival were recorded. Overall survival was defined as the time from surgery to death, while disease-free survival was measured as the time from surgery until the first tumor recurrence or death from any cause. Patients alive or lost to follow-up were censored at the date of last follow-up in survival analysis. Deaths from nondisease-related causes were censored in the analysis of recurrence patterns. The patients were followed up every 3 months for the first 2 years and every 6 months thereafter. Follow-up information was available over 3 years postoperatively or at the date of death.

### Statistical analysis

Statistical analyses were performed using IBM SPSS Statistics (version 25.0 Inc., Chicago, IL, USA) and the R programming language (version 4.0.2, Vienna, Austria). Chi-square or Fisher’s exact test was used for comparisons of categorical data between the two groups. Comparisons of continuous variables were made by two-tailed t test or Wilcoxon rank-sum test. Overall survival curves were calculated using the Kaplan–Meier method, and the log-rank test was used to compare the differences between survival curves. Univariate and multivariate analyses were performed with the Cox proportional hazards regression model. Prognostic factors were determined with respect to both clinical and statistical significance. Statistically significant variables (*P* < 0.10) were entered into the multivariate analysis. Patterns of recurrence were based on univariable Cox regression analysis of disease-free intervals. Restricted cubic spline was used to evaluate the relationship between continuous measures of CRM and hazard ratio of overall survival. Using this method provided greater flexibility for modeling and evaluating the complex relation between survival and variables (14, 15).

## Results

### Baseline characteristics

We screened a total of 89 patients in this study. All patients received neoadjuvant chemoradiotherapy. The clinicopathological characteristics of both cohorts are summarized in **Table 1**. Among various factors, lymphovascular invasion was significantly associated with CRM status (P = 0.033). The patients were classified into two groups, including 50 patients with CRM of 1 mm or less (0 < CRM ≤ 1 mm) and 39 patients with CRM greater than 1 mm (CRM>1 mm).

**Table 1:** Baseline characteristics.

Characteristic	0< CRM ≤1 mm (n=50)	CRM >1 mm (n=39)	*P*
Age, mean ± SD	62.22 ± 8.51	60.62 ± 8.09	0.370
Gender, n (%)			0.726
Female	4 (4.5%)	4 (4.5%)	
Male	46 (51.7%)	35 (39.3%)	
Tumor location, n (%)			0.509
Lower	23 (25.8%)	17 (19.1%)	
Middle	23 (25.8%)	21 (23.6%)	
Upper	4 (4.5%)	1 (1.1%)	
pN stage, n (%)			0.130
N0	18 (20.2%)	21 (23.6%)	
N1	16 (18%)	13 (14.6%)	
N2	9 (10.1%)	4 (4.5%)	
N3	7 (7.9%)	1 (1.1%)	
Differentiation, n (%)			0.667
G1	4 (4.5%)	3 (3.4%)	
G2	30 (33.7%)	27 (30.3%)	
G3	16 (18%)	9 (10.1%)	
LVI, n (%)			0.033
No	38 (42.7%)	37 (41.6%)	
Yes	12 (13.5%)	2 (2.2%)	
PI, n (%)			0.153
No	22 (24.7%)	24 (27%)	
Yes	28 (31.5%)	15 (16.9%)	
TRS, n (%)			0.103
TRS 1	1 (1.1%)	3 (3.4%)	
TRS 2	34 (38.2%)	31 (34.8%)	
TRS 3	15 (16.9%)	5 (5.6%)	
Adjuvant therapy, n (%)			0.967
Yes	23 (25.8%)	19 (21.3%)	
No	27 (30.3%)	20 (22.5%)	

CRM, circumferential resection margin; SD, standard deviation; LVI, lymphovascular invasion; PI, perineural invasion; TRS, tumor regression score according to the modified Ryan scoring system.

### Prognostic impact of CRM status

**Figure 1:**
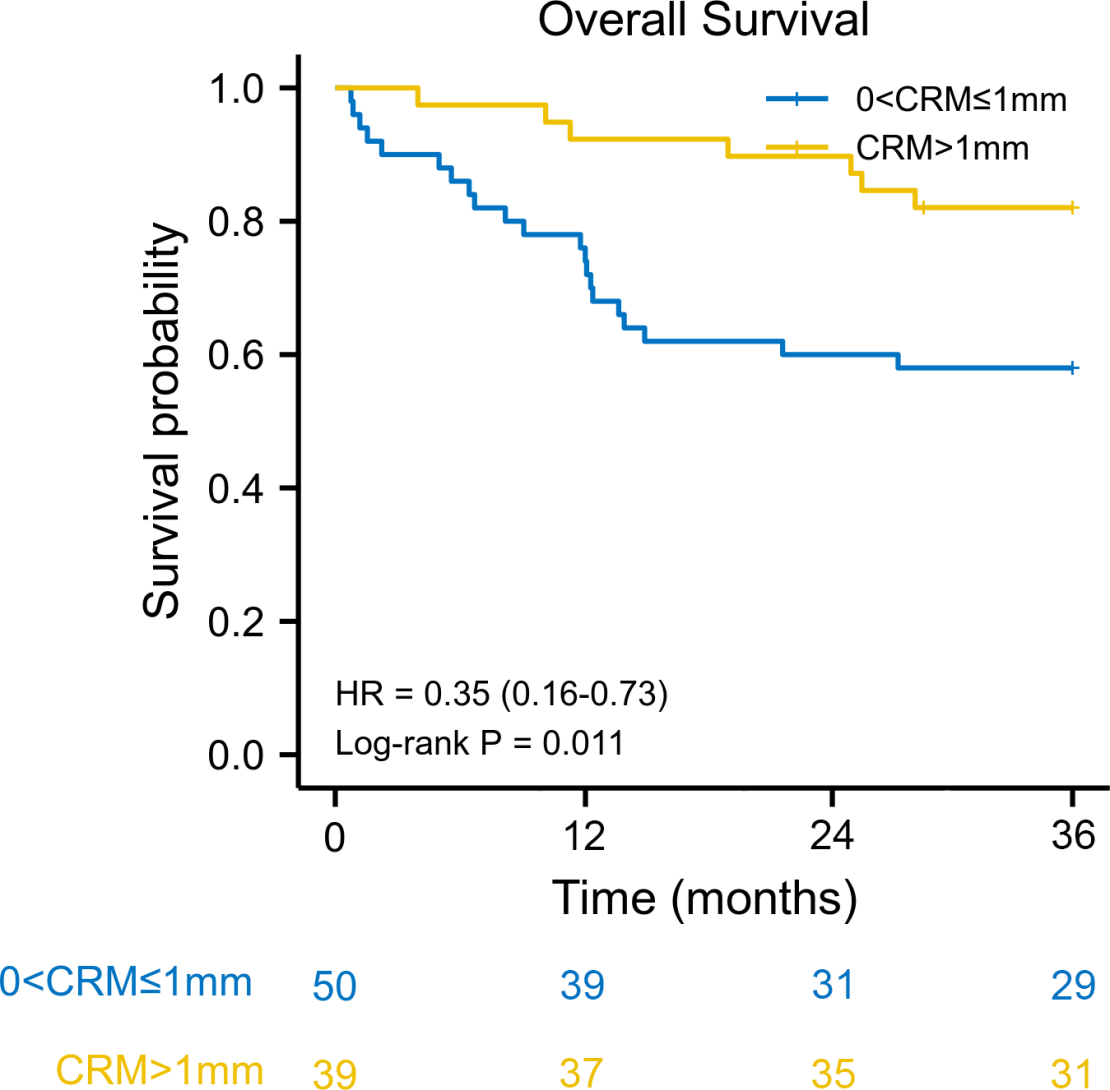
Kaplan–Meier overall survival curve according to circumferential resection margin. CRM, circumferential resection margin; HR, hazard ratio.

**Figure 2:**
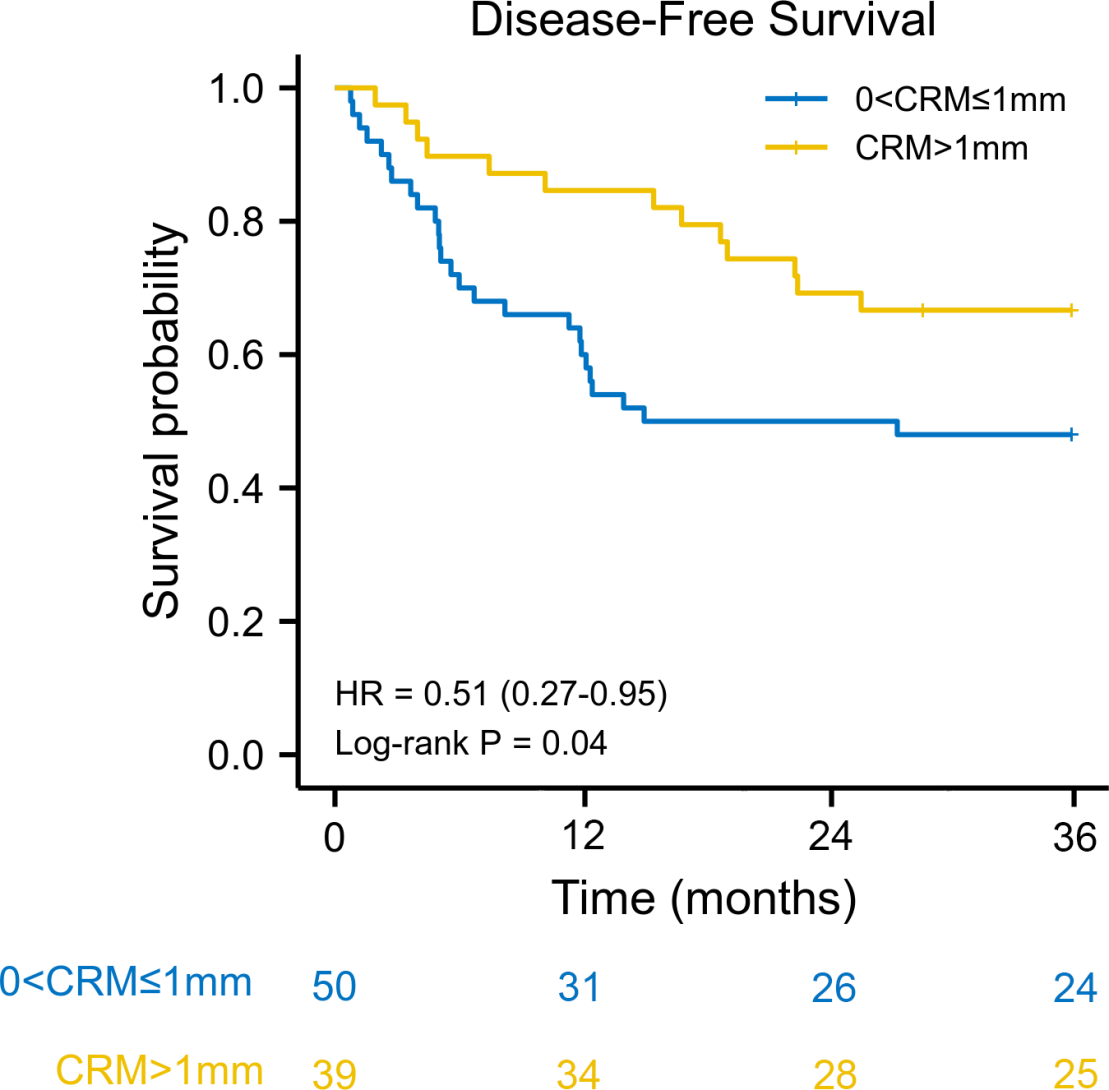
Kaplan–Meier disease-free survival curve according to circumferential resection margin. CRM, circumferential resection margin; HR, hazard ratio.

We used the reverse Kaplan–Meier method to calculate the median follow-up time. The median follow-up time was 42.9 (interquartile range [IQR] 42.0–43.9) months for the entire cohort. The estimated 3-year overall survival was 77.0% (95% CI: 63.5–92.3) for patients with CRM greater than 1 mm, compared with 57.1% (95% CI: 63.5–92.3) for patients with CRM of 1 mm or less. Kaplan–Meier survival analysis revealed that patients with a CRM greater than 1 mm had better overall survival than patients with a CRM of 1 mm or less (HR: 0.35, 95% CI: 0.16–0.73, log–rank *P* = 0.011) (**Figure 1**). The 3-year disease-free survival was 61.6% (95% CI: 48.3–79.2) for patients with CRM greater than 1 mm and 46.3% (95% CI: 33.8–63.7) for patients with CRM of 1 mm or less. There was a significant difference in disease-free survival between the two groups (HR: 0.51, 95% CI: 0.27–0.95, log–rank *P* = 0.040) (**Figure 2**).

A CRM greater than 1 mm was associated with better overall survival in univariate Cox regression (HR: 0.35, 95% CI: 0.15–0.84, log–rank *P* = 0.018). When adjusted for pN stage (P < 0.001) and lymphovascular invasion (P = 0.076), a CRM greater than 1 mm was an independent protective prognostic factor in multivariate analysis (HR: 0.40, 95% CI: 0.17–0.96, log–rank *P* = 0.039). In addition, a CRM greater than 1 mm was correlated with better disease-free survival in univariate analysis (HR: 0.50, 95% CI: 0.26–0.98, log–rank *P* = 0.044). However, a CRM greater than 1 mm did not have a significant impact on disease-free survival in multivariate analysis (HR: 0.53, 95% CI: 0.27–1.05, log–rank *P* = 0.069) (**Table 2**).

**Table 2:** The relation of circumferential resection margins with overall survival and disease-free survival using Cox regression analysis.

	Overall Survival						Disease-Free Survival				
Characteristics	Univariate analysis			Multivariate analysis			Univariate analysis			Multivariate analysis	
	HR (95% CI)	*P*		HR (95% CI)	*P*		HR (95% CI)	*P*		HR (95% CI)	*P*
Age	0.98 (0.94-1.03)	0.556					0.96 (0.92-1.00)	0.064		0.96 (0.92-1.01)	0.137
Gender											
Male	Ref						Ref				
Female	0.81 (0.19-3.43)	0.780					0.80 (0.24-2.60)	0.712			
ypN status											
N0	Ref			Ref			Ref			Ref	
N+	1.45 (1.16-2.04)	<0.001		1.32 (1.07-1.80)	0.006		1.71 (1.35-2.17)	<0.001		1.51 (1.05-2.16)	0.025
Differentiation											
G1	Ref						Ref				
G2/G3	0.77 (0.32-1.82)	0.551					1.62 (0.56-4.65)	0.371			
LVI											
Absence	Ref			Ref			Ref			Ref	
Presence	2.17 (0.92-5.13)	0.076		0.93 (0.37-2.34)	0.894		2.69 (1.30-5.56)	0.007		1.43 (0.60-3.39)	0.412
PI											
Absence	Ref						Ref				
Presence	0.77 (0.36-1.63)	0.502					1.21 (0.64-2.26)	0.552			
CRM											
0<CRM ≤ 1 mm	Ref			Ref			Ref			Ref	
CRM>1 mm	0.35 (0.15-0.84)	0.018		0.40 (0.17-0.96)	0.039		0.50 (0.26-0.98)	0.044		0.53 (0.27-1.05)	0.069

LVI, lymphovascular invasion; PI, perineural invasion, CRM, circumferential resection margin; HR, hazard ratio; CI, confidence interval.

### Recurrence impact of CRM status

We also evaluated the patterns of recurrence for both groups in this study. A total of 42 (47.2%) patients showed disease recurrence in the entire cohort. In the group with CRM greater than 1 mm, 13 patients had disease recurrence, of whom 8 had locoregional recurrence and 7 had distant recurrence (2 patients had mixed recurrence). In the group with a CRM of 1 mm or less, 29 patients had disease recurrence, of whom 19 had locoregional recurrence and 19 had disease recurrence (9 patients had mixed recurrence) (**Table 3**). Compared with a CRM of 1 mm or less, a CRM greater than 1 mm had significantly less locoregional recurrence (HR: 0.34, 95% CI: 0.14–0.86, log–rank *P* =0.022), less distant recurrence (HR: 0.35, 95% CI: 0.15–0.84, log–rank *P* = 0.018), and less overall recurrence (HR: 0.44, 95% CI: 0.23-0.85, log–rank *P* = 0.015).

**Table 3:** Comparison of recurrence patterns between the two groups.

Recurrence	CRM >1 mm (n = 39)	0< CRM ≤1 mm(n = 50)	HR (95% CI)	*P*
Locoregional	8 (20.5%)	19 (38%)	0.34 (0.14-0.86)	0.022
Distant	7 (17.9%)	19 (38%)	0.35 (0.15-0.84)	0.018
Overall	13 (33.3%)	29 (58%)	0.44 (0.23-0.85)	0.015

CRM, circumferential resection margin; HR, hazard ratio; CI, confidence interval.

### Definition of optimal CRM cut point

**Figure 3:**
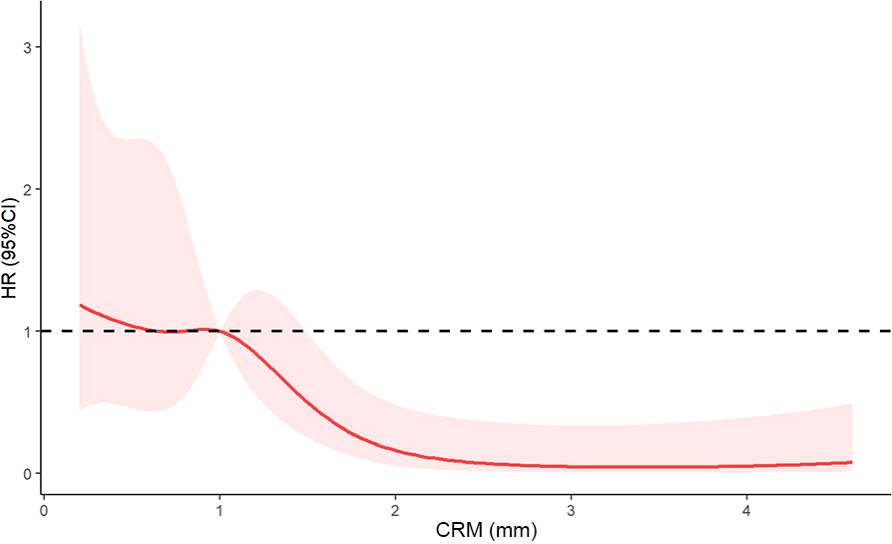
Restricted cubic spline curve of the relationship between circumferential resection margin and hazard ratio of overall survival. The solid red line indicates the hazard ratio, and shaded areas represent the 95% CI. The optimal circumferential resection margin cut point was 1 mm (HR = 1). CRM, circumferential resection margin; HR, hazard ratio; CI, confidence interval.

The association between the hazard ratio of overall survival and continuous measures of CRM using restricted cubic spline curve was shown in **Figure 3**. A nonlinear association between CRM and the hazard ratio of overall survival was noted. A CRM of 1 mm or less had an adverse impact on overall survival (HR < 1). The curve was steep between 1 and 2.5 mm and reached its plateau above 2.5 mm. These results indicated that a longer CRM increased the survival benefit. The optimal circumferential resection margin cut point was 1 mm (HR = 1).

## Discussion

The results of our study elucidated that CRM status had a significant impact on survival and recurrence in esophageal squamous cell carcinoma after neoadjuvant chemoradiotherapy. A CRM greater than 1 mm had significantly better survival and less recurrence than a CRM of 1 mm or less. Simply put, the standard from the Royal College of Pathologists could be more accurate for tumor staging and predicting prognosis for patients with esophageal squamous cell carcinoma who underwent neoadjuvant chemoradiotherapy. Our findings supported that total mesoesophageal excision with adequate CRM could contribute substantially to patient esophageal cancer survival (16). Moreover, adjuvant therapy may be considered for patients with a CRM of 1 mm or less. In a literature review, our results parallel the results of rectal cancer resection after neoadjuvant therapy (17–19). However, Liu et al. reported that the CRM status was less prognostic for esophageal squamous cell cancer after neoadjuvant therapy (20). More high-quality studies might be required to address this topic. Patients who received neoadjuvant therapy may have local advanced disease, a CRM greater than 1 mm could potentially protect the patients from a high risk of recurrence due to the minimal residual disease in induced fibrosis (11, 21, 22).

Furthermore, this study specifically described the patterns of recurrence in both groups. We concluded that a CRM greater than 1 mm had significantly less locoregional recurrence, less distant recurrence, and less overall recurrence than a CRM of 1 mm or less. These results parallel the findings of previous studies on esophageal adenocarcinoma post neoadjuvant chemotherapy (23). Nevertheless, Ghadban et al. (24). indicated that CRM had no effect on recurrence in esophageal cancer with primary surgery. They also suggested investigating the possible impact of neoadjuvant therapy with regard to improved resectability.

Whether tumor location may have an impact on tumor resectability and CRM status remains unclear. Anatomically, the tumors at lower third of esophagus was more easily to be resected radically by surgeons without increasing morbidity, while the situation becomes much more complex with the tumors at middle third of esophagus as they are in close distance to important structures. However, no significant correlation between the tumor location and CRM status was found in our univariate analysis (P = 0.509).

Unexpectedly, a CRM greater than 1 mm did not have a significant impact on disease-free survival in our multivariate analysis (HR: 0.53, 95% CI: 0.27–1.05, log–rank *P* = 0.069). The possible explanation may be that nodal involvement appears to be the most important predictive factor in disease-free survival (25, 26).

Several studies have explored the optimal cut point of CRM. For example, Haneda et al. (8) found that a CRM of 0.6 mm can be the optimal cutoff value. However, non-neoadjuvant therapy population was also included in this study, which may have introduced confounding factors. Notably, a study conducted by Hulshoff et al. (12) showed that the optimal cutoff value was 0.3 mm for the neoadjuvant chemoradiotherapy group. However, their methods of CRM cutoff value determination were not easy to use, and the short follow-up time of 2 years limited the interpretation. In our study, we utilized restricted cubic spline curves to flexibly model and visualize the association between the hazard ratio of overall survival and continuous measures of CRM. We determined the optimal CRM cut point at 1 mm. Our results supported the standards of the Royal College of Pathologists.

The strengths of this study included that only patients who received neoadjuvant chemotherapy were included in our analysis. Moreover, details on locoregional recurrence, distant recurrence and time interval were recorded during the period of follow-up. Thus, the patterns of recurrence were available for both groups. In addition, to better visualize the association between the hazard ratio of overall survival and continuous measures of CRM, restricted cubic spline models were constructed in this study.

Our study also has some limitations. First, it is a retrospective study with inherent flaws. Second, the small sample size warrants further investigation with larger samples. Finally, esophageal squamous cell carcinoma remains the most common histological type in Asia, while adenocarcinomas dominate North America and Europe. The prognostic impact of CRM on esophageal adenocarcinoma requires further investigation.

## Conclusion

We recommend that the optimal CRM criteria be >1 mm. Unfavorable CRM (0 < CRM ≤ 1 mm) should be considered as tumor infiltrated with respect to more recurrence and mortality. A more radical resection with adequate CRM could benefit survival in patients with esophageal squamous cell carcinoma after neoadjuvant therapy.

## Data availability statement

The raw data supporting the conclusions of this article will be made available by the authors, without undue reservation.

## Ethics statement

The studies involving human participants were reviewed and approved by the Ethics Committee of West China Hospital, Sichuan University. Written informed consent for participation was not required for this study in accordance with the national legislation and the institutional requirements.

## Author contributions

L-QC and Y-MG conceptualized the study, revised the manuscript and supervised the study. Y-MG, Y-SY and W-LK collected the data, drafted the manuscript and made the figures. Q-XS, H-LZ, W-PW, YY, and G-WC revised the manuscript.. All authors contributed to the article and approved the submitted version.

## Funding

This research was supported by National Natural Science Foundation of China (82000514), Key Projects of Sichuan Provincial Department of Science and Technology (2021YFS0222), 1•3•5 project for disciplines of excellence–Clinical Research Incubation Project, West China Hospital, Sichuan University (2018HXFH020), Regional Innovation and Collaboration projects of Sichuan Provincial Department of Science and Technology (2021YFQ0026), and National Natural Science Foundation Regional Innovation and Development (U20A20394).

## Conflict of interest

The authors declare that the research was conducted in the absence of any commercial or financial relationships that could be construed as a potential conflict of interest.

## Publisher’s note

All claims expressed in this article are solely those of the authors and do not necessarily represent those of their affiliated organizations, or those of the publisher, the editors and the reviewers. Any product that may be evaluated in this article, or claim that may be made by its manufacturer, is not guaranteed or endorsed by the publisher.
